# Pollock: fishing for cell states

**DOI:** 10.1093/bioadv/vbac028

**Published:** 2022-05-13

**Authors:** Erik P Storrs, Daniel Cui Zhou, Michael C Wendl, Matthew A Wyczalkowski, Alla Karpova, Liang-Bo Wang, Yize Li, Austin Southard-Smith, Reyka G Jayasinghe, Lijun Yao, Ruiyang Liu, Yige Wu, Nadezhda V Terekhanova, Houxiang Zhu, John M Herndon, Sid Puram, Feng Chen, William E Gillanders, Ryan C Fields, Li Ding

**Affiliations:** 1 Department of Medicine, Washington University in St. Louis, St. Louis, MO 63110, USA; 2 McDonnell Genome Institute, Washington University in St. Louis, St. Louis, MO 63108, USA; 3 Department of Surgery, Washington University in St. Louis, St. Louis, MO 63110, USA; 4 Siteman Cancer Center, Washington University in St. Louis, St. Louis, MO 63110, USA

## Abstract

**Motivation:**

The use of single-cell methods is expanding at an ever-increasing rate. While there are established algorithms that address cell classification, they are limited in terms of cross platform compatibility, reliance on the availability of a reference dataset and classification interpretability. Here, we introduce Pollock, a suite of algorithms for cell type identification that is compatible with popular single-cell methods and analysis platforms, provides a set of pretrained human cancer reference models, and reports interpretability scores that identify the genes that drive cell type classifications.

**Results:**

Pollock performs comparably to existing classification methods, while offering easily deployable pretrained classification models across a wide variety of tissue and data types. Additionally, it demonstrates utility in immune pan-cancer analysis.

**Availability and implementation:**

Source code and documentation are available at https://github.com/ding-lab/pollock. Pretrained models and datasets are available for download at https://zenodo.org/record/5895221.

**Supplementary information:**

Supplementary data are available at *Bioinformatics Advances* online.

## 1 Introduction

The use of single-cell methods continues to expand at ever-increasing rates (Tsoucas and Yuan, 2017; Wang and Navin, 2015). Single-cell RNA-sequencing (scRNA-seq) has the capacity to quantify a variety of cell states and biological variability within cells. However, the identification of these cell states for specific datasets is often challenging. In many single-cell workflows, cells are clustered and manually annotated based on known marker gene expression, which can be time consuming and introduce annotator biases. Furthermore, these marker genes are usually derived from bulk RNA-seq data, which can be affected by additional issues, such as limitations of signature matrices used in deconvolution (Aliee and Theis, 2021).

Multiple algorithms for annotating single-cell data currently exist, but are limited in various respects. For example, many single-cell integration methods, such as Seurat (Hao *et al.*, 2020), Scanpy (Wolf *et al.*, 2018) and SingleR (Aran *et al.*, 2019), require a reference dataset for the transfer of cell type annotations, which is not always available or feasible to obtain. Additionally, access to these methods is typically limited to one language (typically R or Python), or modality (API or command line tool), which limits their usability. Perhaps the main scientific impediment is that most current methods do not typically report which features most impact cell classifications, which could provide meaningful insight into biological variation between cells.

Here, we present Pollock, a single-cell classifier aimed at addressing the above concerns. Pollock is an end-to-end, fully differentiable deep learning framework that pairs a variational autoencoder (VAE) with an attached classification head to make cell type predictions. It is highly versatile, being available as a command line tool with Seurat and Scanpy integration, a Python library, and can be installed in containerized form via Docker. To allow for easier pan-disease and pan-tissue analyses, Pollock also ships with a library of pretrained cancer type specific and agnostic models that were trained on expertly curated single-cell data. That is, Pollock pretrained models are ready to ‘plug and play’, with no additional annotation or training required. These pretrained models were fitted on manually curated and annotated single-cell data from eight different cancer types spanning three single-cell technologies: scRNA-seq, single nuclei RNA-seq (snRNA-seq) and single nuclei ATAC-seq (snATAC-seq). Conversely, Pollock allows for the training of custom classification models if an annotated reference single-cell dataset is available. Pollock also provides feature importance scores that allow for cell type classifications to be traced back to the genes influencing a particular cell type classification, further promoting biological interpretability. These scores could allow for new, technology-specific biomarker discovery. We demonstrate the utility of Pollock by applying it in a pan-cancer single-cell immune analysis.

## 2 Methods

### 2.1 Pollock framework

**Fig. 1.**
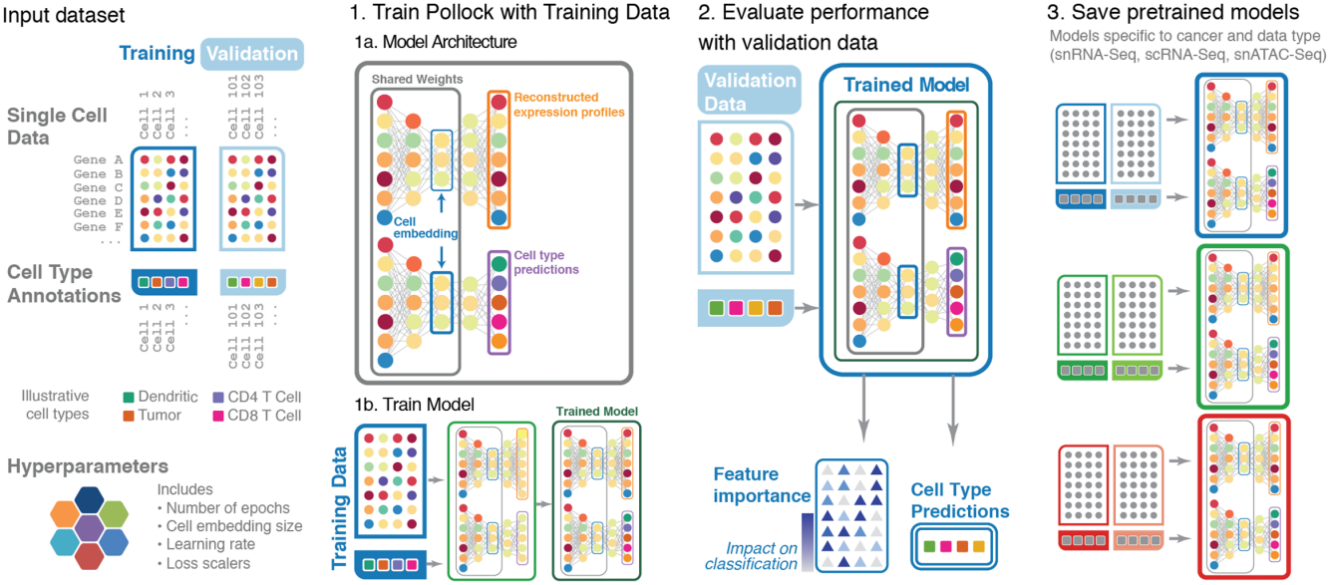
Pollock overview schema. Overview of Pollock model architecture, training, cell type prediction and pretrained models usage. During training, single-cell inputs are split into training and validation sets. (**1a** and **b**) A VAE with a classification head is fit with the training partition of the single-cell data. The model is trained with contributions from three loss functions: KL divergence loss on the latent embedding, ZINB gene expression reconstruction loss and cross-entropy loss on the cell type predictions. (**2**) Evaluation metrics are then computed on a validation set of withheld single-cell data. In addition to cell type prediction, Pollock also outputs feature importance’s for the input features of each predicted cell. (**3**) Following the training, Pollock models are saved and can be used for cell type inference at a later date

Pollock is an end-to-end, fully differentiable VAE (Kingma and Welling, 2013) with an attached classification head. In general, the Pollock model creation pipeline can be broken down into three main steps: (i) dimensionality reduction and cell type classification, (ii) validation and (iii) model saving (Fig. 1).

Dimensionality reduction algorithms are integral to most single-cell workflows (Sun *et al.*, 2019). This step transforms multi-feature, high dimensional single-cell data into a low dimensional manifold of fewer, information-dense features. Typically, this is done with linear methods such as PCA (Lever *et al.*, 2017), nonlinear graph-based methods such as UMAP (McInnes *et al.*, 2018) or t-SNE (Van der Maaten and Hinton, 2008) or a combination of the two. However, an emerging body of literature suggests that neural network-based methods are a better alternative to traditional dimensionality reduction approaches (Lopez *et al.*, 2018). In particular, VAEs preserve local and global structure in the low dimensional manifold (Grønbech *et al.*, 2020; Lopez *et al.*, 2018). VAEs have also demonstrated an ability to generalize well between single-cell datasets (Lotfollahi *et al.*, 2019), making them an attractive option, since single-cell classifiers must address variations that exist in different input datasets. Further, VAEs using a zero-inflated negative binomial (ZINB) loss function parameterized by mean, dispersion and dropout probability to model gene expression reconstruction have been shown to better address issues intrinsic to single-cell data, such as dropout and over-dispersion (Eraslan *et al.*, 2019; Tian *et al.*, 2021).

The gene expression matrix of all cells in a dataset is denoted as ***m*** and the expression profile of cell *x* in the matrix is ***m_x_***. Now, define the conditional distribution P(***m_x_****|****z_x_***) as the probability that a cell ***m_x_*** is drawn from a latent representation stored in vector ***z_x_***. We use a VAE to model P(***m_x_****|****z_x_***), which allows us to obtain an embedding representing each cell’s position on the low dimensional manifold in matrix ***m***. Simultaneously, we condition a classification head on the latent representation ***z_x_*** that is responsible for producing cell type annotations (Fig. 1.1a). To aid the user in the biological interpretation of Pollock’s cell type predictions, it provides interoperability scores that are derived via axiomatic attribution from the Integrated Gradients algorithm (Sundararajan *et al.*, 2017). The interpretability score represents an overall impact score for each feature in the input dataset for each predicted cell. Trained models can then be validated and saved for ‘plug and play’ cell state classification at a later date (Fig. 1.2, 3).

**Fig. 2.**
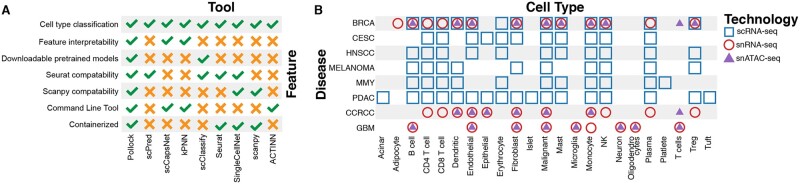
Pollock feature comparison and benchmarking dataset overview. (**A**) Comparison of Pollock features against features implemented in other popular single-cell classification tools. (**B**) Datasets used for benchmarking and the training of disease-specific models

### 2.2 Model implementation

Implementation of the VAE architecture was inspired by scGen (Lotfollahi *et al.*, 2019) for the problem of modeling cellular response to perturbations and scDCC for scRNA-seq clustering (Tian *et al.*, 2021) (Supplementary Fig. S1). Briefly, this is a neural network having both an encoder (embedding component) and a decoder (reconstruction component) that work together in an unsupervised fashion to learn how subject data are generated. Specifically, the network derives an approximation, *Q*, to the true posterior generating function, *P*, for the output, given the input. The encoder and decoder are mirrors of one another, with the encoder consisting of a feature input layer of the same size as the number of genes in the single-cell input, followed by two fully connected layers of size 512 and 128, respectively. Each of these layers is followed by a rectified linear unit (ReLU) activation function. The size of the latent cell embedding layer is 64. Single-cell inputs are normalized to total transcript count, logged and converted to standard *Z*-scores (normalized by standard deviation and mean centered) prior to input into the model. The loss for the VAE portion of the network is the sum of two parts: the ZINB loss of the dropouts, dispersions and means calculated from the raw count inputs and their respective decoder produced values, as implemented in Tian *et al*. (2021), and the conventional β-weighted Kullback–Leibler (KL) distance between *P* and *Q*.

Additionally, a classification head takes as input the latent embedding produced by the VAE and is trained in parallel with the VAE. The classification head consists of a linear layer (ReLU activation) of size 64, followed by a linear layer (Softmax activation) of size equal to the number of possible predicted cell types. The resulting probabilities are compared with manual cell type annotations via cross-entropy loss. The three loss functions are then summed to produce the overall loss used to train the network in the following manner:

$$L_{\mathrm{overall}}=\gamma_{kl}L_{kl}+\gamma_{\mathrm{zinb}}L_{\mathrm{zinb}}+\gamma_{\mathrm{clf}}L_{\mathrm{clf}}$$

where *L*_overall_ is the overall loss and *γ*_kl_, *γ*_zinb_ and *γ*_clf_ are weights for the respective KL divergence (*L*_kl_), ZINB gene expression reconstruction (*L*_zinb_) and cross-entropy cell type classification (*L*_clf_) losses. The model is implemented in the PyTorch Python library (Paszke *et al.*, 2017).

### 2.3 Feature interpretability

We built on previous work to produce explainable outputs via axiomatic attributions. Specifically, we used Integrated Gradients (Sundararajan *et al.*, 2017) to produce a feature importance score for each gene in each predicted cell. In the case of most expression datasets, the features scored are genes. The feature scores, *E*_model_, are constructed by taking the absolute value of attributions calculated by the Captum (Kokhlikyan *et al.*, 2020) implementation of Integrated Gradients. The value of the feature importance score corresponds to the magnitude of impact for that particular input feature on the predicted cell type classification.

### 2.4 Benchmarking preparation and comparison

We used three data types for benchmarking; scRNA-seq was used for pancreatic ductal adenocarcinoma (PDAC), melanoma, multiple myeloma (MMY), breast cancer (BRCA), head and neck squamous cell carcinoma (HNSCC) and cervical squamous cell carcinoma (CESC); snRNA-seq was used for BRCA, clear cell renal cell carcinoma (CCRCC) and glioblastoma multiforme (GBM), and snATAC-seq was used for BRCA, CCRCC and GBM (Supplementary Table S1). Both types of RNA-seq data were maintained as raw, unnormalized counts and normalized according to the documentation for each respective method used for benchmarking. Data were generated and processed as in Zhou *et al.* (2021) and Liu *et al.* (2021). For benchmarking, gene activity, as computed by Signac (Stuart, 2020), was used for snATAC-seq datasets. Gene activity was used for benchmarking because (i) some of the selected benchmarking tools expect input at the gene level and (ii) better performance was seen with gene activity than using peaks directly as input features (Supplementary Fig. S2). Datasets were annotated using cluster assignment based on known markers in the literature (Supplementary Table S2) and are available for download from the CERN Zenodo open-access repository at https://zenodo.org/record/5895221.

We evaluated Pollock against six established cell type classification methods (Supplementary Table S3), namely two popular reference-based approaches, Seurat and Scanpy and four additional top-performing methods from Ma *et al.* (2021) and Abdelaal *et al.* (2019): SingleCellNet (Tan and Cahan, 2019), ACTINN (Ma and Pellegrini, 2020), Linear Support Vector Machines (SVM-linear) and a Multi-layer Perceptron (MLP). For running benchmarks, we set the following Pollock parameters: *γ*_kl_ = 0.001, *γ*_zinb_ = 0.5, *γ*_clf_ = 1.0, epochs = 20, batch size = 64, embedding size = 64 and learning_rate = 1e−4. ACTINN was benchmarked using default parameters. Performance metrics are not available for ACTINN on scRNA-seq MMY and Melanoma datasets due to tool runtime errors. SingleCellNet was run with its own recommended parameters: nTopGenes = 100, nRand = 100, nTrees = 1000, nTopGenePairs = 100, Stratify = True and limitToHVG = True. The linear SVM and MLP were implemented as in Ma *et al.* (2021). Training and validation cells were identical for all tools. Specifically, the training dataset was generated by randomly selecting the number of cells for each cell type as Min(500, 0.8 × total number of cells for that cell type). The validation dataset was then generated by selecting Min(500, 0.2 × total number of cells for cell type) cells that were not part of the training dataset for each cell type. To ensure robustness of results due to random sampling, we also performed inter-dataset validation on five randomly selected folds generated by the criteria described above. Performance of the methods was compared using the standard *F*1 metric, which is the harmonic mean of precision and recall.

Robustness to dropout in prediction datasets was also measured. To simulate datasets with higher dropout rates, additional dropout was computationally added to each scRNA-seq validation dataset. For each dataset, a random selection of 0%, 20%, 40%, 60%, 80% and 90% of positions in the gene expression matrix were set to zero. Performance of Pollock on each of the resulting datasets was compared using the *F*1 metric.

### 2.5 Pretrained models

We have pretrained and packaged models within Pollock for the following data and cancer types: PDAC, Melanoma, MMY, BRCA, HNSCC, CESC (scRNA-seq), BRCA, CCRCC, GBM (snRNA-seq) and BRCA, CCRCC, GBM (snATAC-seq gene activity). We also provide generalized models for each of the three data types: scRNA-seq, snRNA-seq and snATAC-seq. These models were trained with single-cell data from all diseases that were available for their given data type. We removed normal epithelial cells from the training data for the generalized models to alleviate conflict with malignant cells from different disease types. Pollock also includes a model trained on annotations from Hay *et al.* (2018) on data from the Human Cell Atlas (HCA) (Regev *et al.*, 2017). All pretrained models were trained with identical hyperparameters to those used in the benchmarking procedure. Models are available for download from the Zenodo repository at https://zenodo.org/record/5895221, with instructions and documentation on usage available from GitHub at https://github.com/ding-lab/pollock.

### 2.6 Pan-immune pathway analysis

We applied Pollock for pan-immune analysis to illustrate its utility for larger pan-disease analyses with scRNA-seq data by annotating immune cell types. Highly specific immune-cell references were generated via cell type assignment based on known expression markers (Supplementary Table S4).

We then excluded all non-immune cells, leaving 17 cell types, subsequently training a Pollock model on these data. The model was then applied to six diseases having available scRNA-seq data (BRCA, CESC, HNSCC, Melanoma, MMY and PDAC). Pollock hyperparameters during training were identical to those used during benchmarking.

Feature interpretability scores were then calculated for Min(500, # of cells) randomly selected cells not present in the training set for each predicted immune cell state (Supplementary Table S5). Differentially weighted genes (DWGs) were identified by testing for enrichment in feature interpretability scores for each cell state versus all other cell states with the Scanpy rank_genes_groups function with default parameters (Supplementary Table S6). Pathway enrichment analysis for gene feature importance scores was done with ToppFun (Chen *et al.*, 2009). Significant GO: Molecular Function pathways were selected based on enrichment of the top 20 DWGs for natural killer (NK), CD8 T cell-proliferating, T regulatory (Treg) and CD8 T cell-exhausted. Top pathways were rank-ordered by their −log10 FDR corrected *P*-values. Overlap with PanglaoDB version 27_Mar_2020 (Franzén *et al.*, 2019) marker gene sets were computed between the genes in PanglaoDB with human support and the top 100 DWGs for each cell state (Supplementary Table S7). If no direct match for cell state in PanglaoDB was found, the next most macrocell state present in the database was used. Percentage overlap between PanglaoDB marker genes and DWGs for a given cell state was calculated as the ratio of number of overlapping genes to the number of genes in the PanglaoDB marker gene set.

## 3 Results

### 3.1 Pollock provides seamless cell classification across computational platforms

Pollock is designed as an automated solution for long and manual single-cell workflows. To support this aim, we implemented a large set of features to aid in cell annotation and in biological interpretability (Fig. 2A). Existing single-cell classification tools, such as Scanpy and Seurat, use label transfer techniques, partially addressing this issue, but they still require a reference dataset that may be impractical or difficult to obtain. Instead, Pollock provides pretrained models that are available immediately for cell type classification, with no manually labeled dataset or additional model training required. These pretrained models are readily downloadable and seamlessly invoked for use. Additionally, the API allows users to train new models on user-annotated single-cell datasets that may contain unique or domain-specific cell types relevant to their areas of research.

Pollock also incorporates existing single-cell analysis toolkits, including Seurat and Scanpy objects. In terms of usage, it is highly versatile. It provides a command line interface (CLI), making it compatible with command line dependent pipelines and workflow management systems, such as Galaxy (Afgan *et al.*, 2018), CWL (Crusoe *et al.*, 2021) and Snakemake (Köster and Rahmann, 2012). Additionally, Pollock has been containerized, and is available as a Docker container that is portable across Linux, Windows and Mac OS operating systems.

### 3.2 Benchmarking against existing models across a variety of disease and single-cell data types

**Fig. 3.**
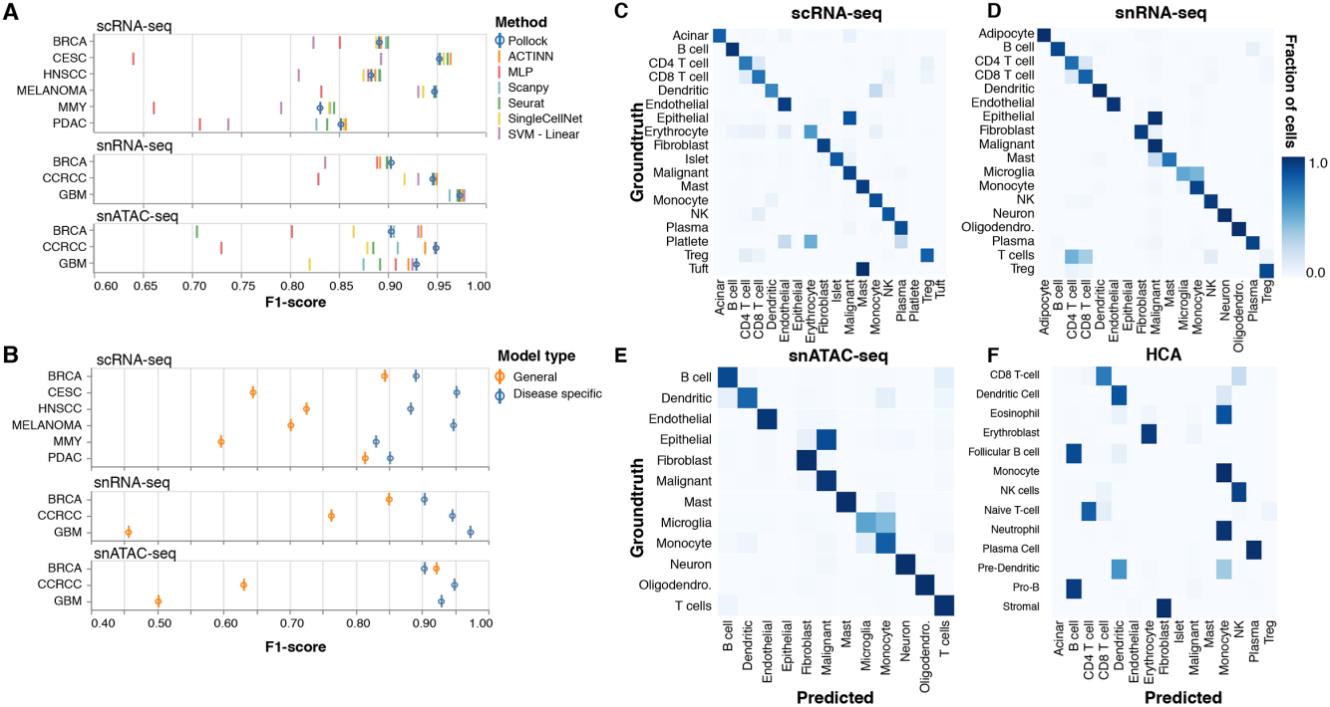
Pollock benchmarking and performance. (**A**) Pollock cell type classification performance (*F*1-score) compared against six established single-cell classification methods for each disease and data type. (**B**) Comparison of Pollock cell type classification performance between disease-specific and generalized models. Confusion matrices showing the overlap of generalized model predicted cell types versus groundtruth cell labels for (**C**–**E**) scRNA-seq, snRNA-seq and snATAC-seq validation datasets and (**F**) a publicly available HCA bone marrow dataset

There are a variety of existing tools for single-cell classification. Here, we compare Pollock against six well-established cell type classification methods. We compared against two popular reference-based approaches, Seurat and Scanpy, as well as four additional top-performing methods from Ma *et al.* (2021) and Abdelaal *et al.* (2019): SingleCellNet (Tan and Cahan, 2019), ACTINN (Ma and Pellegrini, 2020), SVM-linear and an MLP. Training and testing occur on the same dataset, i.e. training data and test samples are sampled from the same cancer and data type. In total, we used 12 single-cell datasets from eight different cancer types (six scRNA-seq, three snRNA-seq and three snATAC-seq) (Fig. 2B). Pollock performance was comparable to existing tools, being within an *F*1-score of 0.02 of the top-performing tool in 11/12 datasets (Fig. 3A, Supplementary Figs S3A–C and S4).

For additional validation, we used a leave-one-out procedure to validate models trained on each cancer type against other datasets of the same data type to test how well Pollock transfers to datasets of different tissue and disease types (Supplementary Fig. S5A–G). Further, to test the robustness of Pollock to prediction datasets with increased dropout rates, we computationally altered validation datasets for each scRNA-seq cancer type (Supplementary Fig. S6A). We find that Pollock performance holds even when up to an additional 60% of dropout is added to the datasets, with performance beginning to decline more steadily at additional dropout rates of >60%.

### 3.3 Training of generalized pan-cancer and HCA models

If they provide pretrained models at all, existing classification tools typically rely on models trained from singleton datasets, resulting in limited ability to annotate cell types from differing tissue and disease types. In addition to its cancer type specific models, we trained generalized Pollock models for scRNA-seq, snATAC-seq and snRNA-seq data types to better generalize its application to new tissue and disease types. Each of these generalized models was trained on the aggregation of single-cell data from all disease types available for the particular data type: scRNA-seq (BRCA, PDAC, CESC, MMY, HNSCC and Melanoma), snRNA-seq (BRCA, GBM and CCRCC) and snATAC-seq (BRCA, GBM and CCRCC).

We assessed the utility of these generalized models by predicting cell types and calculating validation metrics for each cancer types validation dataset when predicted with the generalized model for its corresponding data type. As expected, the generalized models do not perform as well as their disease-specific counterparts due to increased variation introduced into the training dataset by including multiple cancer types. However, the models still are able to classify most cell types correctly, with 8/12 of the models performing within an *F*1-score of 0.30 to their specifically trained counterpart (Fig. 3B). Overall, the best generalizing models were PDAC for scRNA-seq (*F*1-score difference of 0.03) and BRCA for snRNA-seq and snATAC-seq (*F*1-score difference of only 0.06 and 0.02, respectively). Conversely, CESC was the poorest for scRNA-seq (*F*1-score difference of 0.31) and GBM was the poorest for snRNA-seq and snATAC-seq (*F*1-score difference of 0.52 and 0.43, respectively). Additionally, we also predicted cell types on a publicly available HCA (Regev *et al.*, 2017) dataset with annotations by Hay *et al.* (2018). Consistent with known phenotypic differences between cells, the cell types exhibiting the most divergence between model prediction and manual annotation were those with similar phenotypes, such as T cell subsets (CD4, CD8, Treg), and myeloid cells, such as monocytes and dendritic cells (Fig. 3C–F). Additionally, epithelial cells, which were not included in generalized model training sets, are classified as malignant cells which is consistent with the partially epithelial phenotype typically displayed by tumor cells.

### 3.4 Pollock in pan-disease immune analyses

**Fig. 4.**
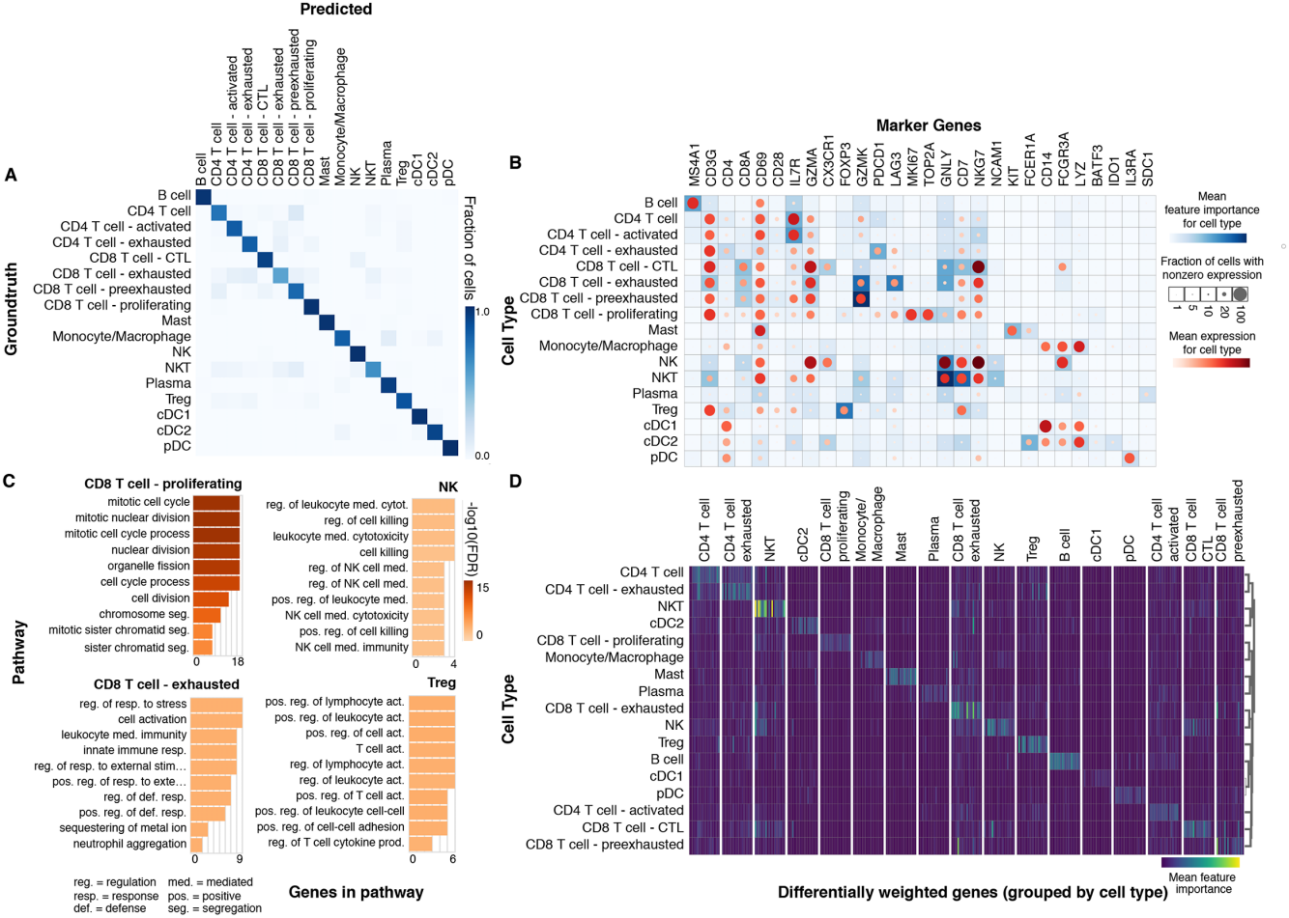
Pollock cell state annotation in a pan-immune atlas. (**A**) Confusion matrix showing overlap of Pollock predicted versus groundtruth cell labels for a scRNA-seq BRCA immune cell state annotated dataset. (**B**) Comparison of Pollock feature importance score and gene expression for literature-based single-cell marker genes. (**C**) Significant GO: Molecular Function pathways enriched in the top 20 DWGs for the following NK/T cell states: NK, CD8 T cell-proliferating, CD8 T cell-exhausted and Treg. Pathways are rank-ordered by their −log10 FDR corrected *P*-values. (**D**) Heatmap displaying feature importance scores for the top 20 DWGs for each immune cell state

To further demonstrate Pollock’s utility, we trained a Pollock model on a scRNA-seq BRCA dataset with detailed immune cell state annotations. Pollock did well in distinguishing the different immune cell states on the breast validation dataset that was withheld during model training, with the majority of cell types having been classified accurately (Fig. 4A). Cell types with the lowest classification accuracy were exhausted CD8 T cells and NKT cells, which were confused most often with other T and NK cell states. Additionally, using Pollock feature interpretability scores, we can identify genes important to the cell type classification, which also show remarkable overlap with known markers in the literature, such as *FOXP3* in regulatory T cells (Roncador, 2005) and *LAG3* and *PDCD1* in exhausted CD8 and CD4 T cells, respectively (Yang *et al.*, 2017; Zhang *et al.*, 2020) (Fig. 4B). This observation indicates that Pollock is focusing on genes known to be associated with specific cell types when making predictions.

Additionally, we identified the most enriched genes for each cell state in terms of their interpretability score, which we designate here as DWGs (Fig. 4C and D; Supplementary Table S6). We performed a pathway enrichment analysis on these genes with GO: Biological Process pathways (The Gene Ontology Consortium, 2000). We highlight the results of the enriched pathways in four T/NK cell states: Treg, CD8 T cell-proliferating, CD8 T cell-exhausted and NK. We see significant enrichment of pathways that are concordant with expected pathways for the cell states, with cell cycle related pathways in the proliferating CD8 T cells, regulation of leukocyte activation in Tregs, cell activation and response to stress in exhausted CD8 T cells and cell killing in NK cells. We further interrogate overlap with known marker genes by comparing Pollock DWGs against the PanglaoDB database of single-cell marker genes (Franzén *et al.*, 2019). We find overlap in the majority of immune cell states with cell types in PanglaoDB, where B, pDC and Treg cells show the most overlap, with 25–29% of Panglao marker genes present in the top DWGs for those cell states (Supplementary Table S7). Less distinct cells in terms of expression profile, such as T cell subsets, show less overlap, potentially due to smaller expression profile differences and the absence of directly overlapping cell states in PanglaoDB (where, for instance, exhausted T cell subsets are not present).

## 4 Discussion

Pollock offers a variety of interfaces (command line and API) while integrating with existing single-cell analysis tools (Seurat and Scanpy). Additionally, existing cell annotation algorithms typically require the use of a reference annotated dataset when making new classifications and do not ship with pretrained models. With Pollock, we include a set of pretrained models that eliminate the usual requirements of either having a reference dataset or training new models for additional datasets. Further, these models score the input features based on their model importance, allowing for greater biological interpretability. These features allow for wide convenience in classifying single-cell data and quickly moving pan-disease atlas projects.

To increase utility to single-cell pipelines, we also trained generalized models for each available training dataset data type (scRNA-seq, snRNA-seq and snATAC-seq). We envision these pretrained models allowing for easier annotation of single-cell datasets due to the removal of the requirement that a user’s single-cell dataset tissue and disease type match with training dataset tissue and disease type. Additionally, in cases where user dataset type matches those used for Pollock training, we provide disease and data type specific models with increased performance for eight cancer types across scRNA-seq, snRNA-seq and snATAC-seq data types.

We showed the utility of pretrained models by demonstrating their efficacy in a pan-cancer single-cell analysis. We also found that a model trained on detailed immune cell states can accurately identify cell states while providing meaningful gene interpretability scores. Overall, results suggest that Pollock should be a useful addition for investigations involving cell type classification.

## Supplementary Material

vbac028_Supplementary_Data
