# Supplementary material for: Pollock: fishing for cell states
Source: Bioinform Adv. 2022 May 13;2(1):vbac028. doi: 10.1093/bioadv/vbac028 (PMC9115775; doi:10.1093/bioadv/vbac028)
Supplement: vbac028_Supplementary_Data [file vbac028_supplementary_data.zip › supplementary_figures.pdf]

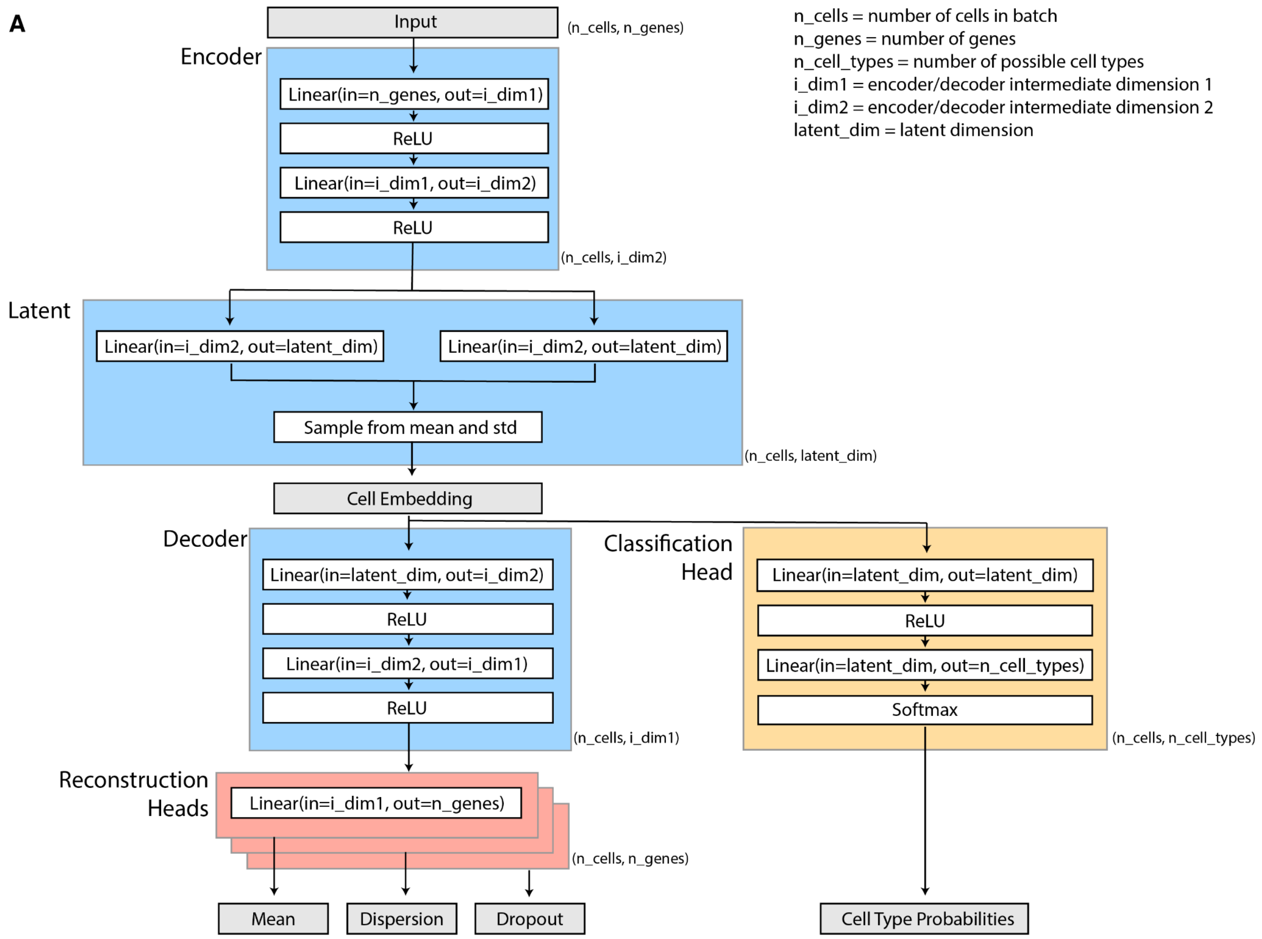

Supplementary Figure 1: Pollock framework

A) Gene expression input is fed into the encoder. Following which, the encoder output is transformed by two linear layers: one producing the latent means and another the latent variances. These means and variances are used to parameterize the distribution the cell embeddings are drawn from. The resulting cell embedding is then simultaneously 1) decoded to produce the mean, dispersion, and dropout to parameterize the ZINB distribution, and 2) input to a cell type classification head to produce cell type classification probabilities.

A

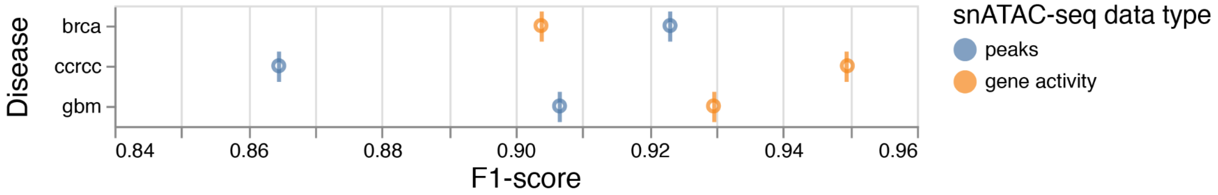

Supplementary Figure 2: Peaks vs. Gene Activity as input for snATAC-seq datasets
A) F1-score of Pollock cell type predictions when trained on peaks and gene activity in BRCA, CCRCC, and GBM snATAC-seq datasets. Peak data was downsampled to the 200k least sparse peaks to alleviate GPU memory pressure.

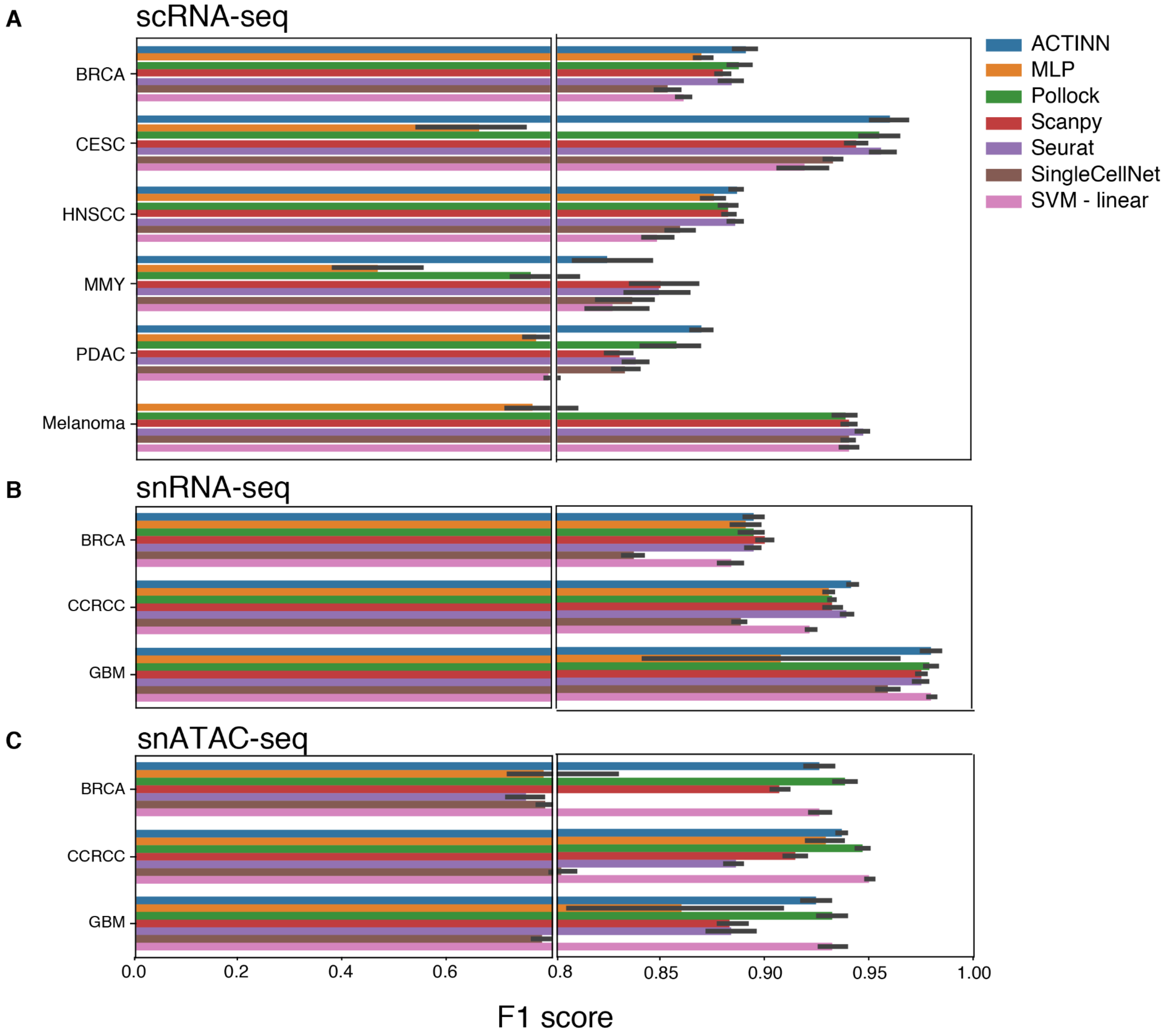

Supplementary Figure 3: Pollock inter-dataset benchmarking
Benchmarking of Pollock and other tools across 5 cross-validation folds for A) scRNA-seq, B) snRNA-seq, and C) snATAC-seq datasets. Error bars show variance in F1-score prediction.

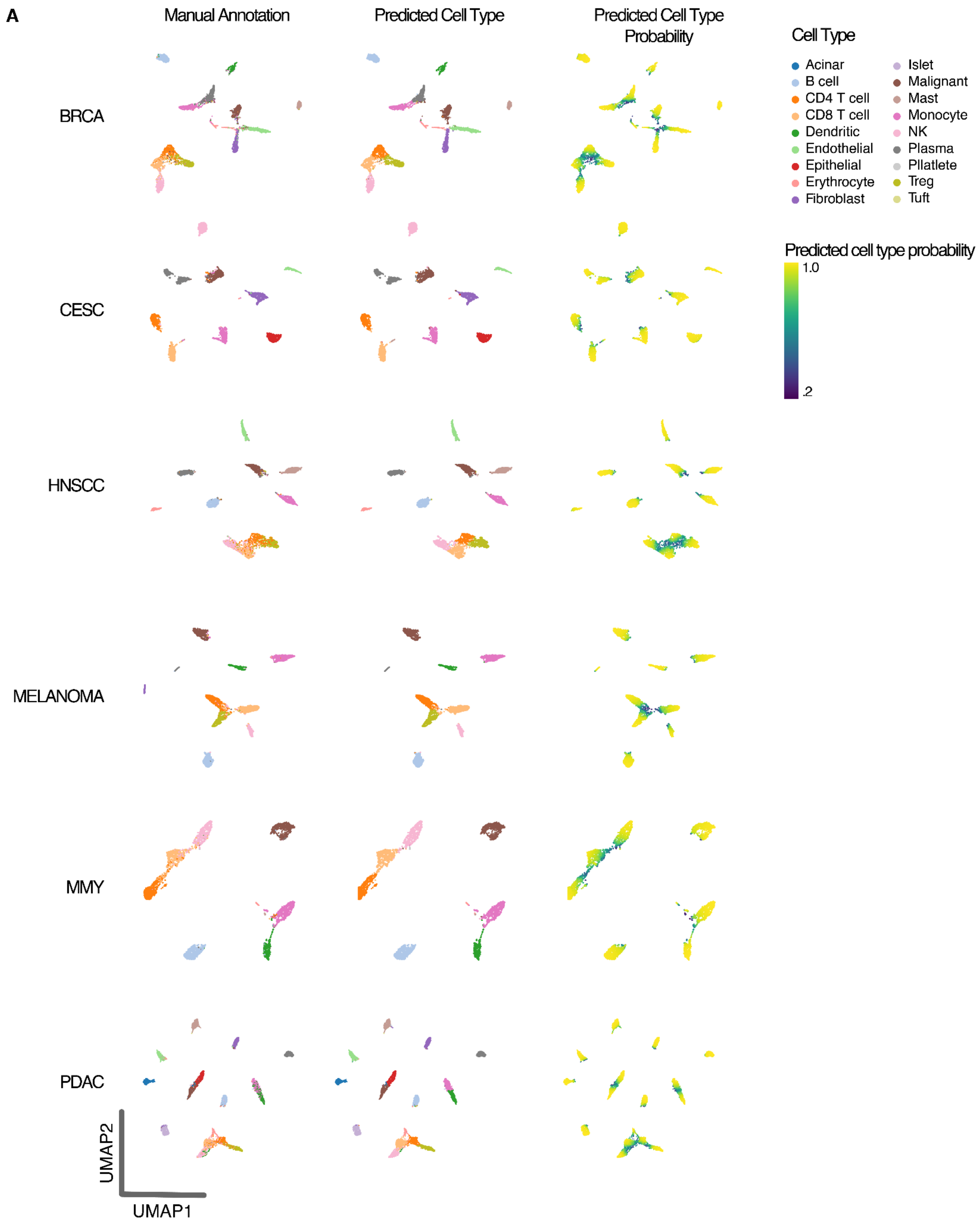

Supplementary Figure 4: Visualization of Pollock cell state predictions for six scRNA-seq datasets
A-F) Visualization of Pollock VAE embedding layer for the validation sets of six different scRNA-seq datasets. The cell embeddings are reduced to a 2-dimensional representation with the UMAP algorithm. Cells are colored from left to right by manual annotation, predicted cell type, and predicted cell type probability.

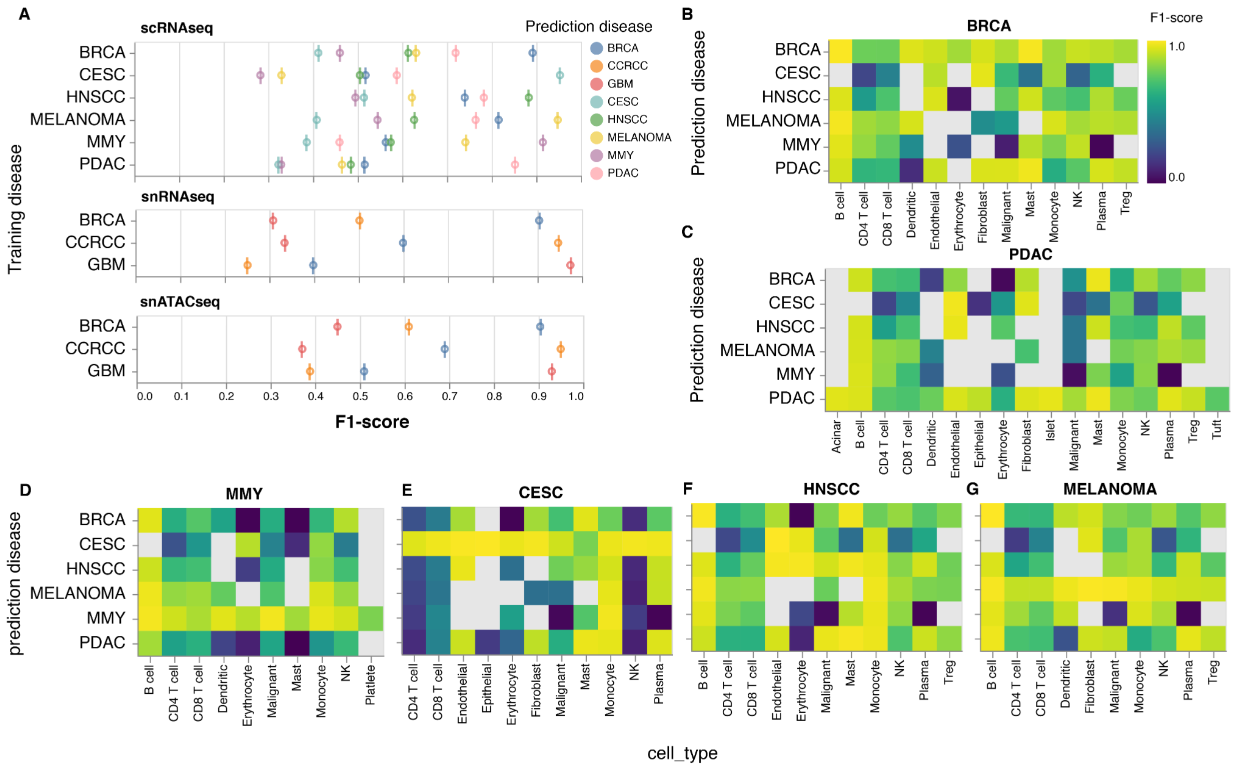

Supplementary Figure 5: Performance of Pollock when applied to cross-disease validation datasets.
A) Performance of Pollock leave-one-out validation, where Pollock was trained on one cancer type and used to predict a differing cancer type for scRNA-seq, snRNA-seq, and snATAC-seq datasets. **B-G)** F1-score by cell type for each training/validation set cancer type pair.

A

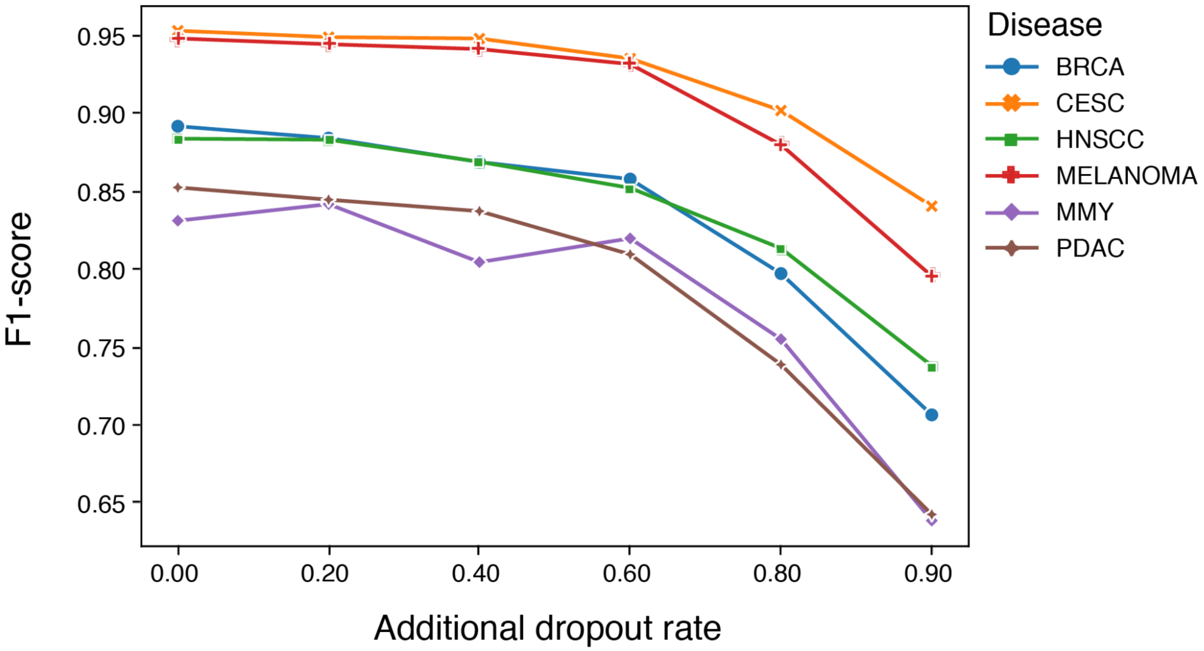

Supplementary Figure 6: Robustness of Pollock to computationally-added prediction dataset dropout.
A) Performance of Pollock pretrained models on validation datasets with varying rates of computationally-added dropout.
