# Supplementary figures and images for: Pollock: fishing for cell states
Source: Bioinform Adv. 2022 May 13;2(1):vbac028. doi: 10.1093/bioadv/vbac028 (PMC9115775; doi:10.1093/bioadv/vbac028)

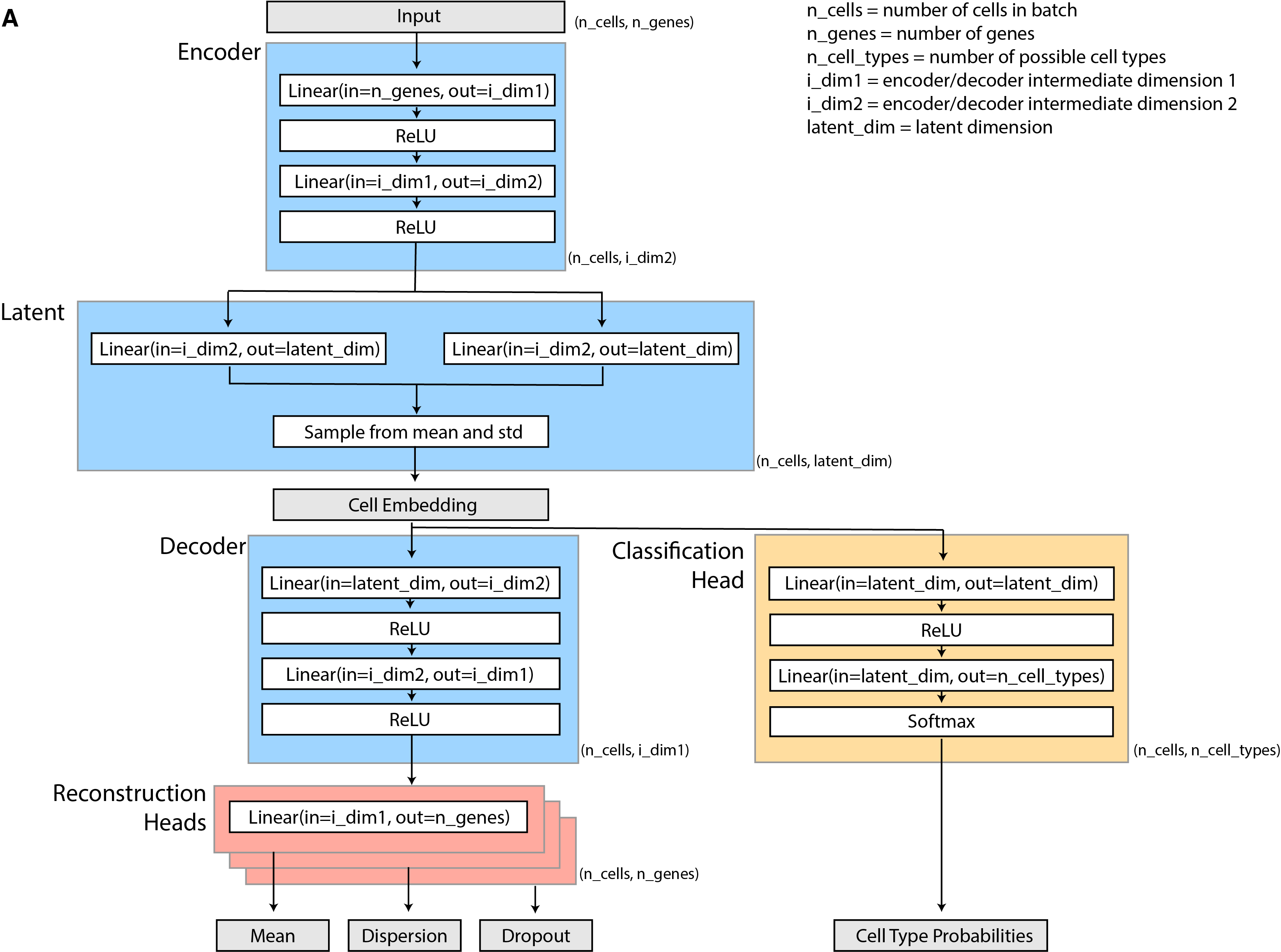

Supplement: vbac028_Supplementary_Data [file vbac028_supplementary_data.zip › pollock_sup_fig_1.tiff]

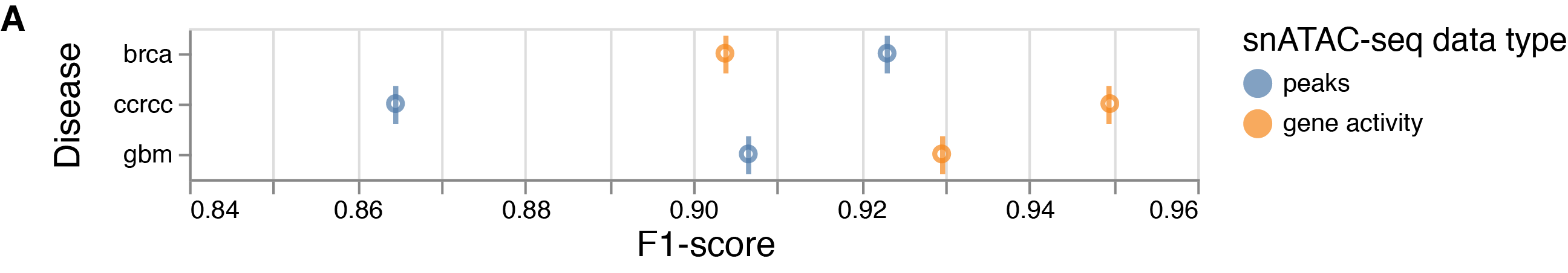

Supplement: vbac028_Supplementary_Data [file vbac028_supplementary_data.zip › pollock_sup_fig_2.tiff]

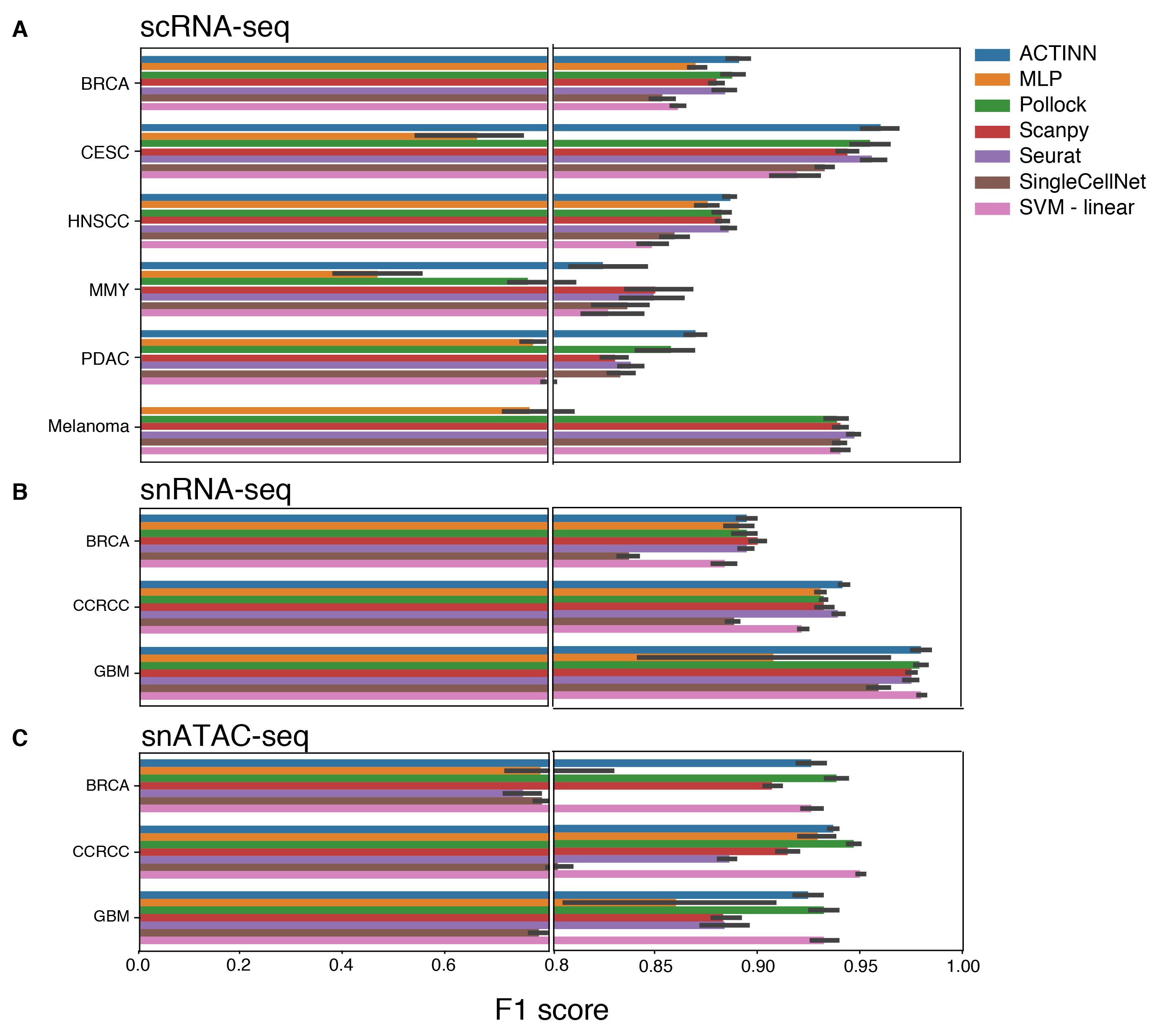

Supplement: vbac028_Supplementary_Data [file vbac028_supplementary_data.zip › pollock_sup_fig_3.tiff]

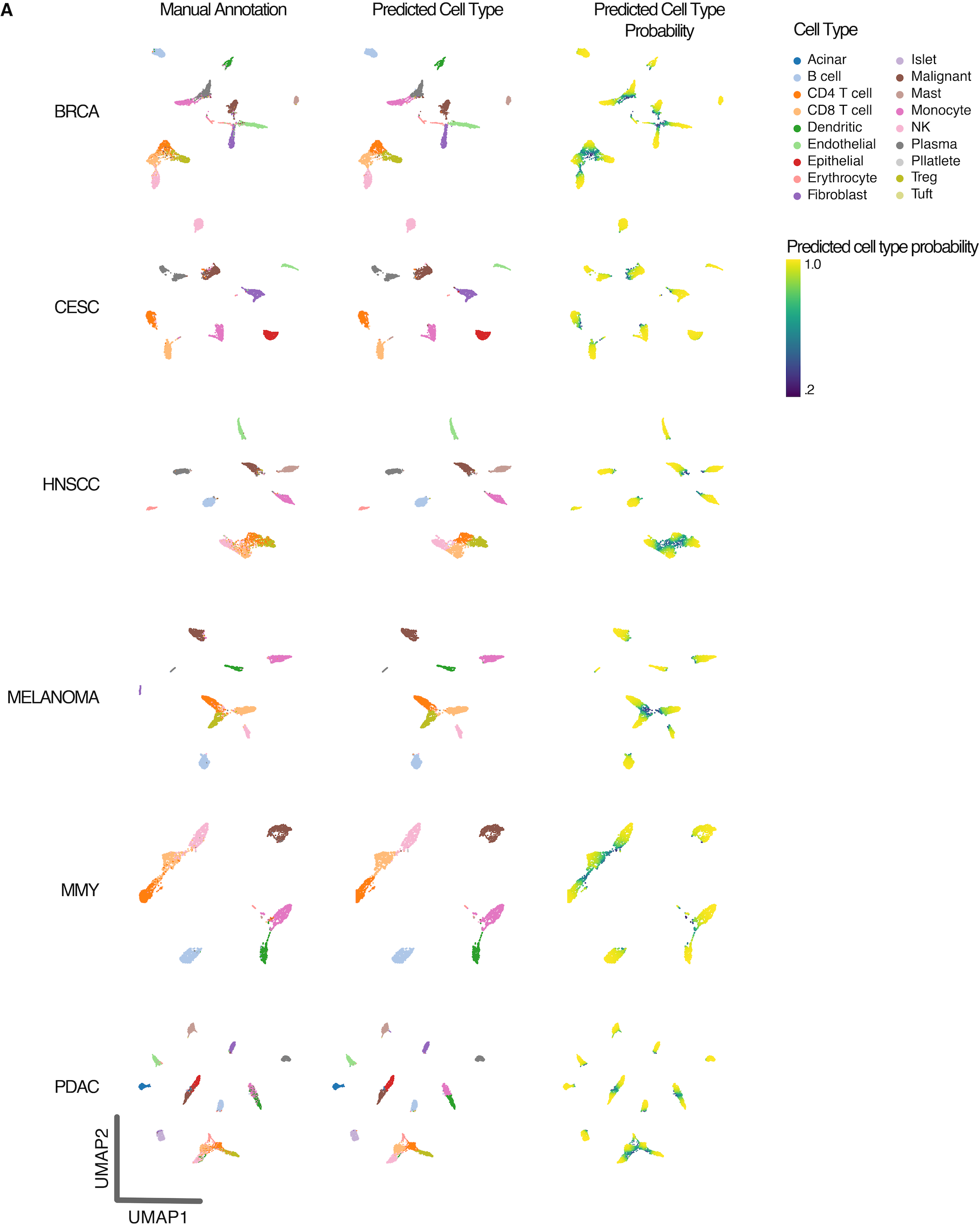

Supplement: vbac028_Supplementary_Data [file vbac028_supplementary_data.zip › pollock_sup_fig_4.tiff]

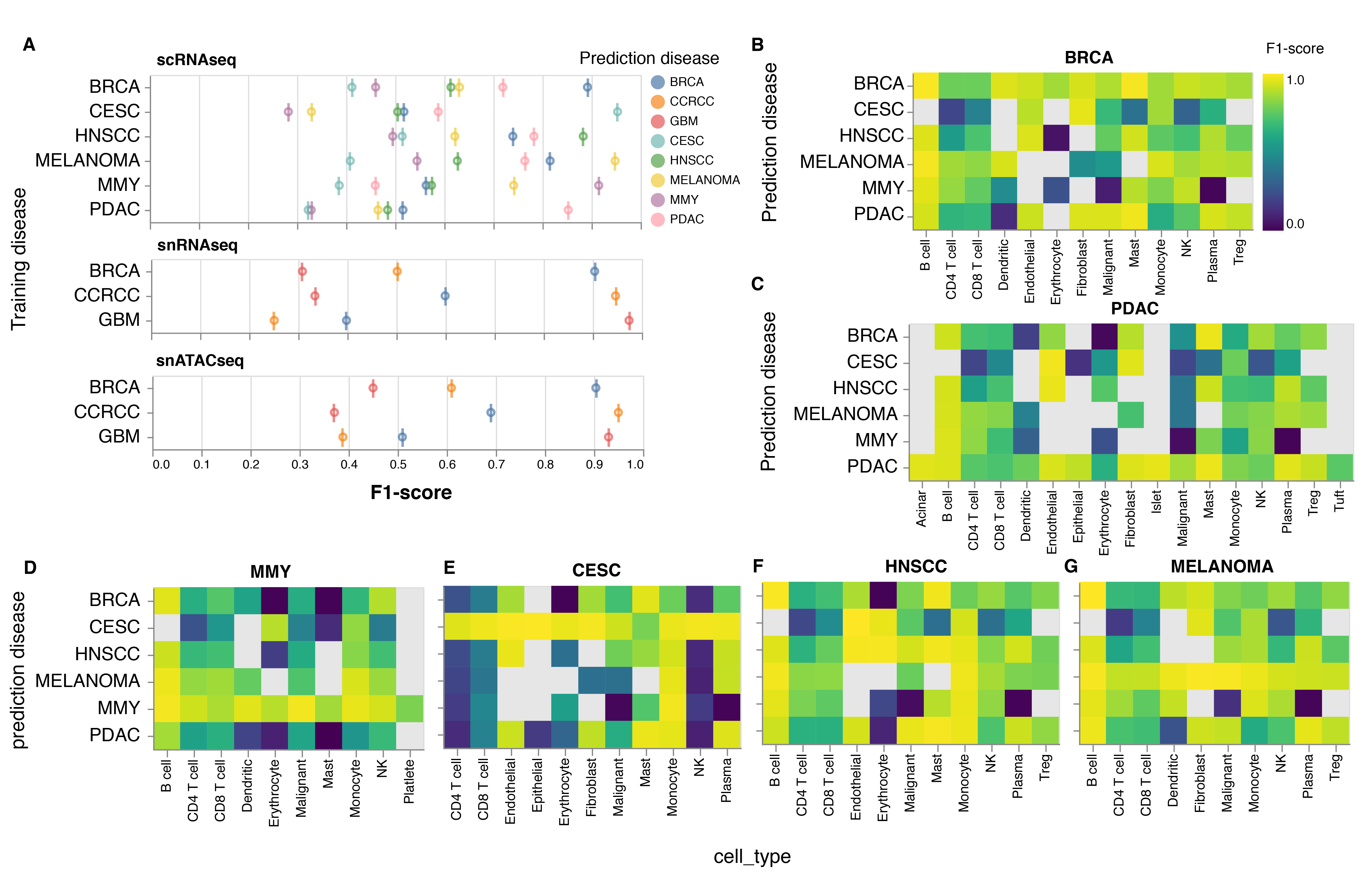

Supplement: vbac028_Supplementary_Data [file vbac028_supplementary_data.zip › pollock_sup_fig_5.tiff]

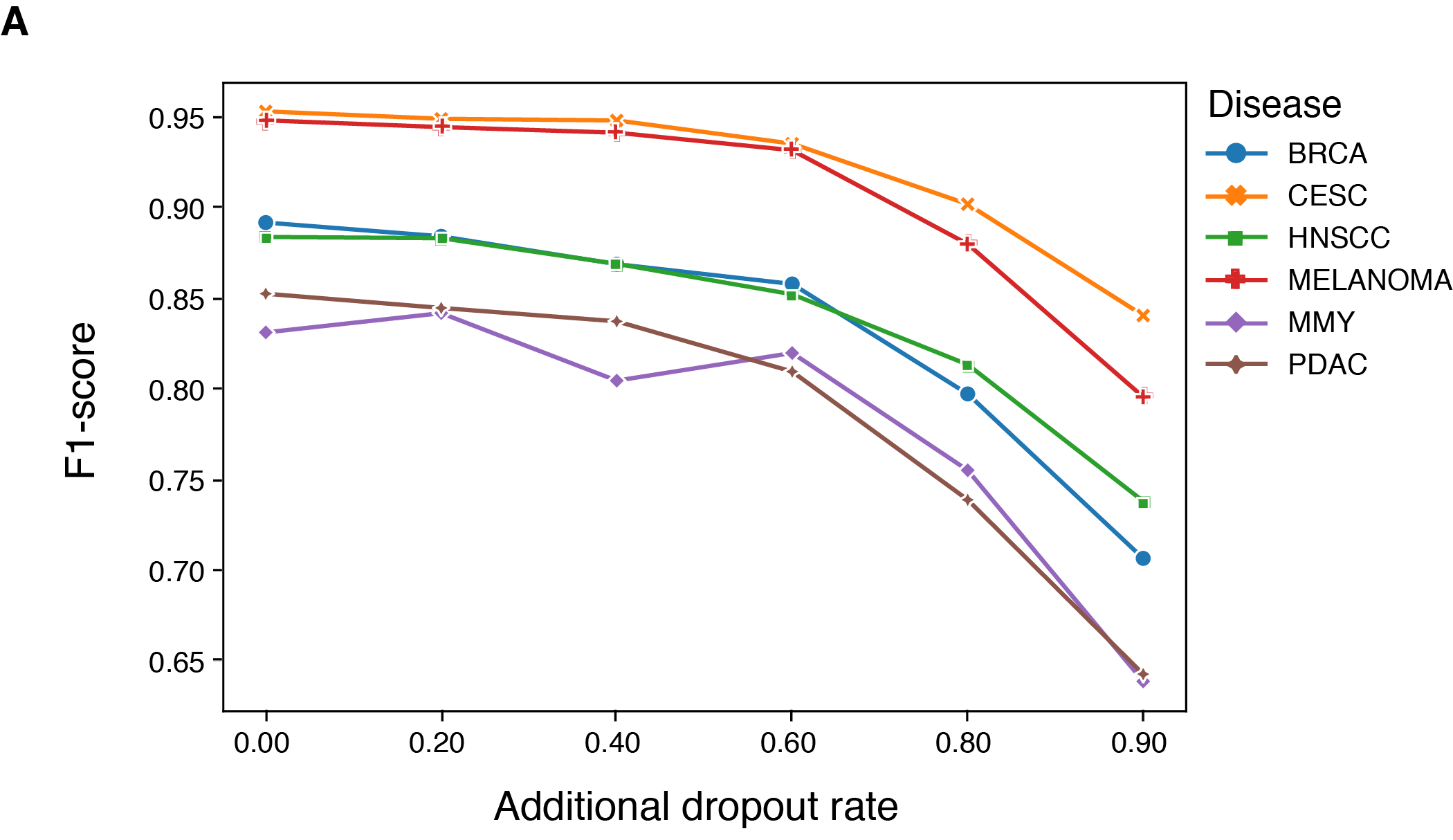

Supplement: vbac028_Supplementary_Data [file vbac028_supplementary_data.zip › pollock_sup_fig_6.tiff]
